# Comparison of Pregnancy Outcomes of Patients Treated With Ondansetron vs Alternative Antiemetic Medications in a Multinational, Population-Based Cohort

**DOI:** 10.1001/jamanetworkopen.2021.5329

**Published:** 2021-04-23

**Authors:** Colin R. Dormuth, Brandace Winquist, Anat Fisher, Fangyun Wu, Pauline Reynier, Samy Suissa, Matthew Dahl, Zhihai Ma, Xinya Lu, Jianguo Zhang, Colette B. Raymond, Kristian B. Filion, Robert W. Platt, Carolina Moriello, J. Michael Paterson

**Affiliations:** 1 Department of Anesthesiology, Pharmacology and Therapeutics, University of British Columbia, Vancouver, British Columbia, Canada; 2 College of Medicine, University of Saskatchewan, Saskatoon, Saskatchewan, Canada; 3 Saskatchewan Health Quality Council, Saskatoon, Saskatchewan, Canada; 4 ICES, Toronto, Ontario, Canada; 5 Center for Clinical Epidemiology, Jewish General Hospital, Lady Davis Institute, Montreal, Quebec, Canada; 6 Department of Epidemiology, Biostatistics and Occupational Health, McGill University, Montreal, Quebec, Canada; 7 Manitoba Centre for Health Policy, University of Manitoba, Winnipeg, Manitoba, Canada; 8 Department of Medicine, Cumming School of Medicine, University of Calgary, Calgary, Alberta, Canada; 9 Department of Medicine, McGill University, Montreal, Quebec, Canada; 10 Department of Pediatrics, McGill University, Montreal, Quebec, Canada; 11 Institute of Health Policy, Management and Evaluation, University of Toronto, Toronto, Ontario, Canada

## Abstract

**Importance:**

Ondansetron is frequently used to treat nausea and vomiting during pregnancy. Although some studies reported important safety signals, few studies have been sufficiently large to assess rare pregnancy outcomes.

**Objective:**

To study the association between ondansetron exposure during pregnancy and the risks of spontaneous abortion, stillbirth, and major congenital malformations.

**Design, Setting, and Participants:**

This is a cohort study conducted in 3 countries, with a meta-analysis. Participants included women and girls aged 12 to 55 years who experienced spontaneous abortion, induced abortion, stillbirth, or live birth between April 2002 and March 2016, as recorded in administrative data from 5 Canadian provinces (British Columbia, Alberta, Saskatchewan, Manitoba, and Ontario), the US IBM MarketScan Research Databases, and the UK Clinical Practice Research Datalink. The statistical analysis was completed in October 2020.

**Exposures:**

Exposure to ondansetron during pregnancy was compared with exposure to other commonly used antiemetics to minimize confounding by indication.

**Main Outcomes and Measures:**

The primary outcome was fetal death, defined as either spontaneous abortion or stillbirth. Secondary outcomes were the 2 components of the primary outcome and major congenital malformations identified during the year after a live birth. Adjusted hazard ratios were estimated using Cox proportional hazards models with time-dependent drug exposures and were adjusted using high-dimensional propensity scores. For major congenital malformations, adjusted odds ratios were estimated from logistic models. Site-level results were pooled using random-effects meta-analysis. Sensitivity analyses considered second-line antiemetic exposure and exposure specifically during 4 to 10 weeks of gestation.

**Results:**

Data from 456 963 pregnancies were included in this study of fetal death (249 787 [54.7%] in Canada, 197 913 [43.3%] in the US, and 9263 [2.0%] in the UK; maternal age, ≤24 years, 93 201 patients [20.4%]; 25-29 years, 149 117 patients [32.6%]; 30-34 years, 142 442 patients [31.2%]; and ≥35 years, 72 203 patients [15.8%]). Fetal death occurred in 12 907 (7.9%) of 163 810 pregnancies exposed to ondansetron, and 17 476 (5.7%) of 306 766 pregnancies exposed to other antiemetics. The adjusted hazard ratios were 0.91 (95% CI, 0.67-1.23) for fetal death with time-dependent ondansetron exposure during pregnancy, 0.82 (95% CI, 0.64-1.04) for spontaneous abortion, and 0.97 (95% CI, 0.79-1.20) for stillbirth. For major congenital malformations, the estimated odds ratio was 1.06 (95% CI, 0.91-1.22). Results of sensitivity analyses were generally consistent with those of the primary analyses.

**Conclusions and Relevance:**

In this large, multicenter cohort study, there was no association between ondansetron exposure during pregnancy and increased risk of fetal death, spontaneous abortion, stillbirth, or major congenital malformations compared with exposure to other antiemetic drugs.

## Key Points

**Question:**

What is the association between ondansetron exposure during pregnancy and the risk of adverse fetal outcomes?

**Findings:**

In this meta-analysis of cohort studies of 456 963 pregnancies in 3 countries, treatment with ondansetron was not significantly associated with increased risk of fetal death, spontaneous abortion, stillbirth, or major congenital malformations compared with other antiemetics.

**Meaning:**

These findings suggest that ondansetron use during pregnancy is not associated with an increased risk of adverse fetal outcomes compared with the use of other antiemetics.

## Teaser

This cohort study examines the association between ondansetron exposure during pregnancy and the risks of spontaneous abortion, stillbirth, and major congenital malformations.

## Introduction

Approximately 80% of women experience nausea and vomiting during pregnancy (NVP),^1^ and approximately 20% to 25% of pregnant women use the 5-HT_3_ receptor antagonist ondansetron off label to treat NVP.^2,3^ Several studies have considered adverse outcomes of ondansetron in pregnancy, but few have considered rarer outcomes such as stillbirth. A notable exception is a study by Pasternak and colleagues,^4^ which reported a nonsignificant reduction in stillbirth among women exposed to ondansetron compared with nonexposure (adjusted hazard ratio [HR], 0.42; 95% CI, 0.10-1.73). In addition, little is known about the safety of ondansetron for treating NVP compared with other prescription antiemetics.

More research is available for ondansetron and congenital malformations; however, studies to date have been comparisons of ondansetron exposure to nonexposure and have shown somewhat conflicting results. Among studies^5,6,7,8,9^ that investigated major congenital malformations overall, odds ratios (ORs) ranged from 0.57 (95% CI, 0.13-2.49)^7^ to 1.04 (95% CI, 0.83-1.30).^8^ Oral-facial clefts and cardiac defects have also been studied in association with ondansetron exposure compared with nonexposure.^6,9,10,11,12^ Estimates in those studies ranged from a risk ratio of 0.95 (95% CI, 0.63-1.43)^9^ to an OR of 2.37 (95% CI, 1.18-4.76).^12^ In studies^5,6,9,10,11,13^ that examined cardiac defects, estimates ranged from a risk ratio of 0.97 (95% CI, 0.86-1.10) for cardiac malformations^9^ to a risk ratio of 2.1 (95% CI, 1.1-4.0) for ventricular septal defects.^13^

Despite signals of harm in some studies, in particular for congenital malformations, evidence regarding the safety of ondansetron in pregnancy remains inconsistent and inconclusive. Health Canada submitted a query (Q16-08) regarding ondansetron and malformations to Canada’s Drug Safety and Effectiveness Network, for which the Canadian Network for Observational Drug Effect Studies (CNODES) undertook a large, multicenter analysis of ondansetron and various adverse pregnancy outcomes. Ondansetron-exposed women in previous studies were largely compared with women who did not use antiemetics, including a study^4^ that reported a protective association between ondansetron and spontaneous abortion. Further research on ondansetron vs other antiemetics in adverse fetal outcomes is thus warranted given that NVP has been associated with a decreased risk of spontaneous abortion.^14^

## Methods

Research ethics board approval was obtained at each site except Ontario, where the requirement was waived. Informed consent was not required by the research ethics boards because all data were deidentified and the study was, therefore, deemed to be of minimal risk to patient privacy. This study follows the Strengthening the Reporting of Observational Studies in Epidemiology (STROBE) reporting guideline.

### Study Design and Data Source

We undertook a multicenter cohort study of women and girls aged 12 to 55 years with a spontaneous abortion, induced abortion, stillbirth, or live birth between April 2002 and March 2016, in the administrative health data from 5 Canadian provinces (British Columbia, Alberta, Saskatchewan, Manitoba, and Ontario social assistance recipients), the IBM MarketScan Research Databases from the US (hereafter referred to as MarketScan), and the United Kingdom’s Clinical Practice Research Datalink (UK CPRD), a clinical database that contains general practitioner practice records that were linked to Hospital Episode Statistics hospitalization data and Office for National Statistics vital statistics information. Information on the databases used in this study is provided in eAppendix 1 in the Supplement. Patients were required to have continuous drug and medical coverage for at least 1 year before any pregnancy outcome. Alberta data were unavailable for analyses of congenital malformations. Ontario data were not used for analyses of fetal death, spontaneous abortion, and stillbirth because of a prespecified requirement of at least 15 ondansetron-exposed pregnancies before applying cohort exclusion criteria.

### Identification of Study Cohort

The study cohort included patients with a dispensation (or prescription in the CPRD) for ondansetron or another antiemetic during pregnancy. We constructed pregnancy episodes where pregnancy onset was defined as the first day of the last menstrual period, estimated by subtracting gestational age (GA) from either the date of birth or the date of fetal death. Availability of GA varied across jurisdictions, with 3% of CPRD, 48% of Canada, and 100% of MarketScan data requiring imputation. GA is routinely collected for in-hospital abortions and births in Canada but is not captured by physician billing data. Where GA at the time of the pregnancy outcome was not available, we used an algorithm developed by Hornbrook et al^15^ to impute GA according to the median GA for each pregnancy event type (spontaneous abortion, induced abortion, stillbirth, or live birth). The date of each pregnancy outcome was then used to define the end of a pregnancy episode, with the pregnancy outcome date minus GA (plus 1 day) defining the estimated date of the last menstrual period. Participants were permitted to contribute multiple pregnancy episodes to the study cohort.

### Identification of Outcome Events

The primary outcome was fetal death, which was a composite of spontaneous abortion and stillbirth (eAppendix 2 in the Supplement). Three secondary outcomes were spontaneous abortion and stillbirth (analyzed separately) and major congenital malformations. Spontaneous abortion and stillbirth were captured through hospital discharge abstracts, emergency department records, vital statistics, and physician service claims during pregnancy. In the CPRD, pregnancy outcomes were identified using the CPRD’s pregnancy register. Each site was included in the outcome-specific analyses if it was anticipated to have at least 15 events among patients with a dispensation for ondansetron during pregnancy, on the basis of an analysis of antiemetic drug use conducted at each site before the study.

Diagnosis and procedure codes used to define outcomes are provided in the eAppendix 3 in the Supplement. In databases other than CPRD, which had a pregnancy register, each inpatient delivery generated a hospital discharge record that included a unique patient identifier (Canada) or family identifier (MarketScan), which was used to link mothers and their infants. This linkage was used for the analysis of congenital malformations, and event ascertainment also included hospital and physician services records for the infant in the 365 days following live birth. Major congenital malformations were defined using an adaptation of the classification schemes developed by other organizations.^16,17,18^ Live births with chromosomal anomalies, genetic syndromes, congenital virus infections, and other anomalies with known causes were excluded from the malformations study cohort.^4^ Our approach was similar to that used in other studies,^19,20,21^ which reported congenital malformations coding in Quebec hospital discharge abstracts and physician service claims that had reasonable positive (78.1%) and negative (94.2%) predictive values.

### Exposure Measurement

We studied patients who used prescription ondansetron or a comparator antiemetic from a community pharmacy during pregnancy. Eligible comparator drugs were diclectin (doxylamine with pyridoxine), metoclopramide, or promethazine. For fetal death and stillbirth, exposure to antiemetic medications was assessed any time during gestation. For spontaneous abortion, exposure was assessed from the beginning of gestation until the occurrence of an outcome or 140 days gestation (20 weeks), whichever occurred first. For major congenital malformations, exposure was assessed during the first 84 days of gestation (12 weeks) only. In sensitivity analyses, exposure for all outcomes was between 29 and 70 days (4 to 10 weeks) of gestation, an assumed period of maximal susceptibility of the fetus to teratogenic effects.

For the primary outcome and for the secondary outcomes of spontaneous abortion and stillbirth, exposure was defined as a time-dependent variable to avoid immortal time bias^22,23^; this was particularly important given the likelihood that ondansetron would be second-line therapy for NVP in Canada, and hence prescribed later during pregnancy than comparators. Using this approach, follow-up began with first use of ondansetron or a comparator drug, and patients were considered exposed until the end of pregnancy, regardless of the quantity or days supply of medication dispensed. Patients who were dispensed a comparator drug followed by ondansetron could contribute person-time to both exposure categories, as defined by the first dispensing dates of the comparator and ondansetron. Once a patient was exposed to ondansetron, she was considered ondansetron-exposed until the end of follow-up. A patient who received only a comparator antiemetic was considered comparator-exposed until the end of follow-up. For major congenital malformations, exposure was defined using a time-fixed approach, in which patients dispensed only a comparator medication were assigned to the comparator group, and those dispensed ondansetron were assigned to the ondansetron group, regardless of whether they also received a comparator. Details of exposure ascertainment methods are provided in eAppendix 4 in the Supplement.

### Sensitivity and Subgroup Analyses

Several sensitivity and subgroup analyses were performed. First, the MarketScan database and the CPRD were excluded from the meta-analyses to generate Canada-only estimates. Further analyses were repeated under the following scenarios: (1) examining only antiemetic exposures occurring during the first 4 to 10 weeks of GA; (2) when a patient became pregnant more than once, restricting the analyses to only the first pregnancy; (3) comparison of second-line ondansetron exposure to second-line comparator exposure, as measured by the second class of antiemetic taken by patients who took more than 1 antiemetic; (4) a matched-pair cohort analysis of siblings to examine potential confounding by genetic and environmental factors; (5) an analysis of cardiac malformations; and (6) an analysis that permitted bidirectional switching between ondansetron and comparator drugs. The sibling analysis was conducted in the British Columbia database, which was the only database where a combination of cohort size and longitudinal mother-infant linkage would permit such an analysis. The sibling cohort included pairs of siblings who were born during the study period and for whom the mother was exposed to ondansetron during 1 pregnancy but not the other. For patients with parity of 3 or higher, priority was given to discordant ondansetron-comparator matches. If more than 1 ondansetron-comparator pair occurred, then 1 was selected at random. Analysis of cardiac malformations was limited to the British Columbia and MarketScan databases because only those databases contained sufficient sample sizes. A model with bidirectional switching was estimated in the largest Canadian database (British Columbia) as a check of our approach in the main analysis that once a patient was exposed to ondansetron, she was considered exposed until the end of pregnancy.

### Statistical Analysis

We used Cox proportional hazards models with time-dependent exposure to estimate HRs for fetal death, spontaneous abortion, and stillbirth. Logistic regression was used to estimate adjusted ORs for major congenital malformations. High-dimensional propensity scores were estimated and included in all models to minimize potential confounding.^24^ In addition to any confounding variables automatically detected by the high-dimensional propensity score algorithm, we required the following potential confounding variables to be included in the high-dimensional propensity score model: calendar year of pregnancy outcome, maternal age, pregnancy history (live births, spontaneous abortions, induced abortions, and stillbirths in the previous 5 years), hospitalization for hyperemesis gravidarum, history of diabetes, immunodeficiency disorders, prior health service use, and claims for prescription drugs suspected to influence risk for adverse pregnancy outcomes. Patients were excluded from analyses if their propensity scores were near the ends of the propensity score distribution where nonoverlap occurred between ondansetron-exposed and comparator-exposed patients. Further information on our propensity score approach is available upon request. Adjusted estimates produced at each CNODES site were pooled using random-effects meta-analysis with inverse variance weighting. The significance threshold was a 95% CI for HRs and ORs that excluded 1.0. Data analyses at each site were conducted using various versions of SAS statistical software (SAS Institute). Meta-analysis was conducted using Review Manager software version 5.3 (The Cochrane Collaboration). Data analysis was completed in October 2020.

## Results

The main analysis of fetal death included 456 963 pregnancies exposed to ondansetron or a comparator antiemetic (249 787 pregnancies [54.7%] in Canada, 197 913 pregnancies [43.3%] in the US, and 9263 pregnancies [2.0%] in the UK; maternal age ≤24 years, 93 201 patients [20.4%]; 25-29 years, 149 117 patients [32.6%]; 30-34 years, 142 442 patients [31.2%]; and ≥35 years, 72 203 patients [15.8%]). Pregnancies exposed to ondansetron or a comparator were identified from a source population of 4 116 424 pregnancies (Table 1), composed of live births (2 733 517 pregnancies [66.4%]), spontaneous abortions (842 112 pregnancies [20.5%]), induced abortions (501 165 pregnancies [12.2%]), and stillbirths (39 630 pregnancies [1%]). A detailed analysis of drug utilization in our study will be provided in a future publication. In brief, the period prevalence of exposure during pregnancy was 4.5% (185 086 pregnancies) for ondansetron and 11.4% (466 693 pregnancies) for other antiemetics. As expected, most antiemetic exposures were during the first trimester. Although the overall prevalence of antiemetic use was similar in the US (16.2% [296 995 pregnancies]) and Canada (19.3% [340 928 pregnancies) by the end of the study period, it was less in the CPRD database (3.6% [13 856]). In Canada and the UK, 3.0% of antiemetic exposures (10 592 pregnancies) involved ondansetron compared with 58.8% (174 494 pregnancies) in the MarketScan database.

**Table 1.** Pregnancies in Included Databases, by Pregnancy Outcome^a^

Pregnancy outcome	Pregnancies, No. (%)							
	Alberta (n = 448 567)	Manitoba (n = 276 654)	MarketScan (n = 1 834 006)	Ontario (n = 115 267)	Saskatchewan (n = 231 287)	British Columbia (n = 823 184)	UK CPRD (n = 387 459)	Total (N = 4 116 424)
Abortion								
Induced	72 415 (16.1)	48 395 (17.5)	122 340 (6.7)	41 381 (35.9)	27 335 (11.8)	182 367 (22.2)	6932 (1.8)	501 165 (12.2)
Spontaneous	73 777 (16.4)	33 939 (12.3)	483 249 (26.3)	12 269 (10.6)	31 891 (13.8)	111 445 (13.5)	95 542 (24.7)	842 112 (20.5)
Birth								
Stillbirth	6658 (1.5)	1369 (0.5)	20 331 (1.1)	638 (0.6)	1303 (0.6)	4459 (0.5)	4872 (1.3)	39 630 (1)
Live	295 717 (65.9)	192 951 (69.7)	1 208 086 (65.9)	60 979 (52.9)	170 758 (73.8)	524 913 (63.8)	280 113 (72.3)	2 733 517 (66.4)
Congenital malformations	NA	18 636 (6.7)	85 146 (4.6)	5140 (4.5)	14 873 (6.4)	39 847 (4.8)	10 011 (2.6)	173 653 (4.2)

Abbreviation: CPRD, Clinical Practice Research Datalink.

^a^ Participating sites were Manitoba (2002-2003 to 2015-2016), British Columbia (2002-2003 to 2015-2016), CPRD and Saskatchewan (2002-2003 to 2015-2016), Ontario (2002-2003 to 2016-2017), MarketScan (2006-2007 to 2015-2016), and Alberta (2009-2010 to 2015-2016).

Baseline characteristics of the study cohort for the primary outcome are presented in Table 2 according to the first antiemetic received, which was ondansetron for 150 197 pregnancies and was another antiemetic for 306 766 pregnancies. There were 13 613 of 163 810 ondansetron-exposed patients overall who received ondansetron after another antiemetic. Treatment time for those pregnancies was counted proportionately in both exposure categories, but events were counted only in the ondansetron category. Ontario was excluded from the analysis of fetal death because of an insufficient number of exposed events. Patients exposed to ondansetron were slightly older than those exposed to a comparator, with those younger than 25 years noticeably less likely to use ondansetron (16.5% of ondansetron-exposed pregnancies) than another antiemetic (22.3% pregnancies exposed only to other antiemetics). Use of ondansetron increased during most of the study period, from 28 pregnancies between 2001 and 2004 to 75 576 pregnancies between 2011 and 2013, before becoming less prevalent between 2014 and 2016, with 23 787 pregnancies. There was a marked difference in pregnancy history between the exposure groups, with patients exposed to ondansetron being less likely to have a history of live birth (2.1% of ondansetron-exposed pregnancies vs 37.4% of pregnancies exposed to other antiemetcis) or history of spontaneous abortion (9.9% of ondanestron-exposed pregnancies vs 19.5% of pregnancies exposed to other antiemetics). This association was attributable primarily to the MarketScan data where, generally, less medical history data were available. The use of various prescription drugs and medical services was variable and at least partly attributable to inclusion of US data, where ondansetron is much more commonly used and patterns of health care utilization are unlike those in Canada.

**Table 2.** Characteristics of Pregnant Patients Exposed to an Antiemetic Medication During Pregnancy^a^

Variable	Pregnancies, No. (%)			
	All databases		Canadian databases only	
	Ondansetron^b^	Comparator	Ondansetron^a^	Comparator
Pregnancies, No.^c^	150 197	306 766	5747	244 040
Alberta	4501 (3.0)	66 851 (21.8)	4501 (78.3)	66 851 (27.4)
British Columbia	687 (0.5)	114 322 (37.3)	687 (12.0)	114 322 (46.8)
Manitoba	96 (0.1)	25 788 (8.4)	96 (1.7)	25 788 (10.6)
Saskatchewan	463 (0.3)	37 079 (12.1)	463 (8.1)	37 079 (15.2)
MarketScan	144 198 (96.0)	53 715 (17.5)	NA	NA
CPRD	252 (0.2)	9011 (2.9)	NA	NA
No. of distinct mothers	148 526 (98.9)	258 101 (84.1)	5457 (95.0)	196 138 (80.4)
Age at conception, y				
≤24	24 722 (16.5)	68 479 (22.3)	943 (16.4)	54 381 (22.3)
25-29	46 062 (30.7)	103 055 (33.6)	2203 (38.3)	83 649 (34.3)
30-34	51 939 (34.6)	90 503 (29.5)	1768 (30.8)	71 689 (29.4)
≥35	27 474 (18.3)	44 729 (14.6)	833 (14.5)	34 321 (14.1)
Calendar year of conception				
2001-2004	28 (0)	28 008 (9.1)	13 (0.2)	26 576 (10.9)
2005-2007	7099 (4.7)	43 355 (14.1)	26 (0.5)	33 167 (13.6)
2008-2010	43 703 (29.1)	93 661 (30.5)	1049 (18.3)	69 281 (28.4)
2011-2013	75 576 (50.3)	109 730 (35.8)	3820 (66.5)	90 495 (37.1)
2014-2016	23 787 (15.8)	32 012 (10.4)	835 (14.5)	24 521 (10.0)
Pregnancy history in the 5 y before conception				
Live birth	3089 (2.1)	114 808 (37.4)	2773 (48.3)	110 529 (45.3)
Spontaneous abortion	14 832 (9.9)	59 686 (19.5)	1369 (23.8)	53 235 (21.8)
Induced abortion	1388 (0.9)	25 218 (8.2)	625 (10.9)	24 815 (10.2)
Stillbirth	417 (0.3)	2502 (0.8)	88 (1.5)	2246 (0.9)
Hospitalization for hyperemesis gravidarum	915 (0.6)	6288 (2.0)	334 (5.8)	2792 (1.1)
Comorbidities in the 5 y before conception				
Diabetes	6583 (4.4)	32 042 (10.4)	835 (14.5)	29 726 (12.2)
HIV and other immunodeficient states	53 (0)	2000 (0.7)	43 (0.7)	1920 (0.8)
Hospital admissions, No.				
0	140 464 (93.5)	264 468 (86.2)	4814 (83.8)	206 893 (84.8)
1-2	9376 (6.2)	40 191 (13.1)	830 (14.4)	35 348 (14.5)
≥3	319 (0.2)	2107 (0.7)	68 (1.2)	1799 (0.7)
Physician visits, No.				
0	10 116 (6.7)	12 210 (4.0)	144 (2.5)	6064 (2.5)
1-2	25 820 (17.2)	32 908 (10.7)	449 (7.8)	21 438 (8.8)
3-4	28 798 (19.2)	40 375 (13.2)	589 (10.2)	28 654 (11.7)
≥5	85 451 (56.9)	221 273 (72.1)	4553 (79.2)	187 884 (77.0)
Prescriptions in the 365 d before conception, No.				
0	14 879 (9.9)	61 718 (20.1)	990 (17.2)	57 320 (23.5)
1-2	35 295 (23.5)	91 571 (29.8)	1571 (27.3)	78 431 (32.1)
3-4	32 977 (21.9)	63 385 (20.7)	1182 (20.6)	49 393 (20.2)
≥5	67 046 (44.7)	90 092 (29.4)	2004 (34.9)	58 896 (24.1)
Prescriptions within first 41 d after conception				
Proton-pump inhibitors or histamine-2 receptor agonists	10 925 (7.3)	23 109 (7.5)	747 (13.0)	18 022 (7.4)
Nonsteroidal antiinflamatory	26 460 (17.6)	51 754 (16.9)	1170 (20.4)	39 577 (16.2)
Antimigraine	5785 (3.9)	7160 (2.3)	193 (3.4)	4024 (1.6)
In vitro fertilization	18 117 (12.1)	20 274 (6.6)	510 (8.9)	14 315 (5.9)
Oral antibiotics	87 404 (58.2)	150 288 (49.0)	2959 (51.5)	111 802 (45.8)
Immunosuppressive agents	7660 (5.1)	5996 (2.0)	155 (2.7)	2980 (1.2)
Oral corticosteroids	19 301 (12.9)	15 292 (5.0)	244 (4.2)	7514 (3.1)
Antiepileptic agents	7155 (4.8)	10 201 (3.3)	278 (4.8)	6931 (2.8)
Angiotensin-converting enzyme inhibitors or angiotensin II receptor blockers	1180 (0.8)	1625 (0.5)	35 (0.6)	911 (0.4)
Anticoagulants	174 (0.1)	252 (0.1)	6 (0.1)	172 (0.1)
Statins	649 (0.4)	703 (0.2)	18 (0.3)	400 (0.2)
Dermatologicals	3573 (2.4)	5558 (1.8)	112 (1.9)	4380 (1.8)
Pituitary, hypothalamic and sex hormones	17 275 (11.5)	11 415 (3.7)	291 (5.1)	5835 (2.4)
Psycholeptic and psychoanaleptic agents	1306 (0.9)	4773 (1.6)	56 (1.0)	4051 (1.7)
Other potential teratogens	234 (0.2)	2322 (0.8)	57 (1.0)	2208 (0.9)
Metformin	4024 (2.7)	4066 (1.3)	98 (1.7)	2480 (1.0)

Abbreviations: CPRD, Clinical Practice Research Datalink; NA, not applicable.

^a^ Baseline characteristics of the study cohort for the primary outcome of fetal death are shown.

^b^ Patients who received a comparator followed by ondansetron are included in the comparator category. Only patients who received ondansetron as first-line treatment are in the ondansetron category.

^c^ Ontario data are not shown as the primary outcome was not evaluated in that province.

Counts of the primary and secondary outcomes are provided in Table 3. There were 30 383 fetal deaths during follow-up, most of which (26 519 deaths [87%]) were spontaneous abortions. Fetal death occurred in 12 907 (7.9%) of 163 810 pregnancies exposed to ondansetron, and 17 476 (5.7%) of 306 766 pregnancies exposed only to other antiemetics. There were 233 696 pregnancies in our study of congenital malformations, of which 69 605 (29.8%) were exposed to ondansetron. Crude rate ratios for the time-to-event outcomes were, in Canada and the CPRD combined, 0.87 (95% CI, 0.77-0.97) for fetal death, 0.99 (95% CI, 0.87-1.13) for spontaneous abortion, and 1.40 (95% CI, 1.10-1.78) for stillbirth. In the MarketScan database, crude rate ratios were 0.57 (95% CI, 0.54-0.59) for fetal death, 0.56 (95% CI, 0.54-0.58) for spontaneous abortion, and 0.83 (95% CI, 0.75-0.92) for stillbirth. Adjusted HRs from the time-dependent Cox models are shown in the Figure. After combining results from each database using random-effects meta-analysis, ondansetron use in pregnancy was not associated with an increased risk of fetal death (HR, 0.91; 95% CI, 0.67-1.23), spontaneous abortion (HR, 0.82; 95% CI, 0.64-1.04), stillbirth (HR, 0.97; 95% CI, 0.79-1.20), or major congenital malformations (OR, 1.06; 95% CI, 0.91-1.22). In a subgroup analysis of cardiac malformations, the adjusted ORs for malformations were 1.31 (95% CI, 0.75-2.31) in the British Columbia database and 0.81 (95% CI, 0.69-0.96) in the MarketScan database. The risks of other types of major malformations were not modeled separately because of a paucity of events in the Canadian databases.

**Table 3.** Adverse Pregnancy Outcomes in Patients Using Antiemetics

Outcome	Canadian and UK CPRD sites		MarketScan	
	Ondansetron	Comparator^a^	Ondansetron	Comparator^a^
Fetal death				
Pregnancies, No.	8437	253 051	155 373	53 715
Person-wk of exposure, No.	204 638	6 834 731	4 042 209	1 014 566
Events, No.	310	11 897	12 597	5579
Events per 1000 wk of exposure, No.	1.51	1.74	3.12	5.50
Crude rate ratio (95% CI)	0.87 (0.77-0.97)	NA	0.57 (0.54-0.59)	NA
Spontaneous abortion				
Pregnancies, No.	7114	244 492	141 825	46 772
Person-wk of exposure, No.	76 466	3 271 068	1 390 784	358 831
Events, No.	237	10 229	10 967	5086
Events per 1000 wk of exposure, No.	3.10	3.13	7.89	14.17
Crude rate ratio (95% CI)	0.99 (0.87-1.13)	NA	0.56 (0.54-0.58)	NA
Stillbirth				
Pregnancies, No.	7947	217 273	155 431	53 712
Person-weeks of exposure, No.	193 922	5 878 974	4 043 690	1 014 504
Events, No.	70	1517	1632	493
Events per 1000 wk of exposure, No.	0.36	0.26	0.40	0.49
Crude rate ratio (95% CI)	1.40 (1.10-1.78)	NA	0.83 (0.75-0.92)	NA
Major congenital malformations				
Pregnancies, No.	2289	149 697	67 316	14 394
Major congenital malformations, events, No. (%)^b^	206 (9.00)	11 673 (7.80)	5436 (8.08)	1126 (7.82)
Crude odds ratio (95% CI)	1.17 (1.01-1.35)	NA	1.03 (0.96-1.11)	NA
Ventricular septal defects, No. (%)	10 (0.44)	366 (0.24)	228 (0.34)	48 (0.33)
Cardiac defects, No. (%)	20 (0.87)	899 (0.60)	834 (1.24)	213 (1.48)

Abbreviations: CPRD, Clinical Practice Research Datalink; NA, not applicable.

^a^ Patients taking a comparator followed by ondansetron (n = 13 613) have treatment time counted in both exposure categories, but events are counted only in the ondansetron category.

^b^ Except for ventricular septal defects and cardiac defects, small cell sizes precluded reporting of other specific malformations.

**Figure.**
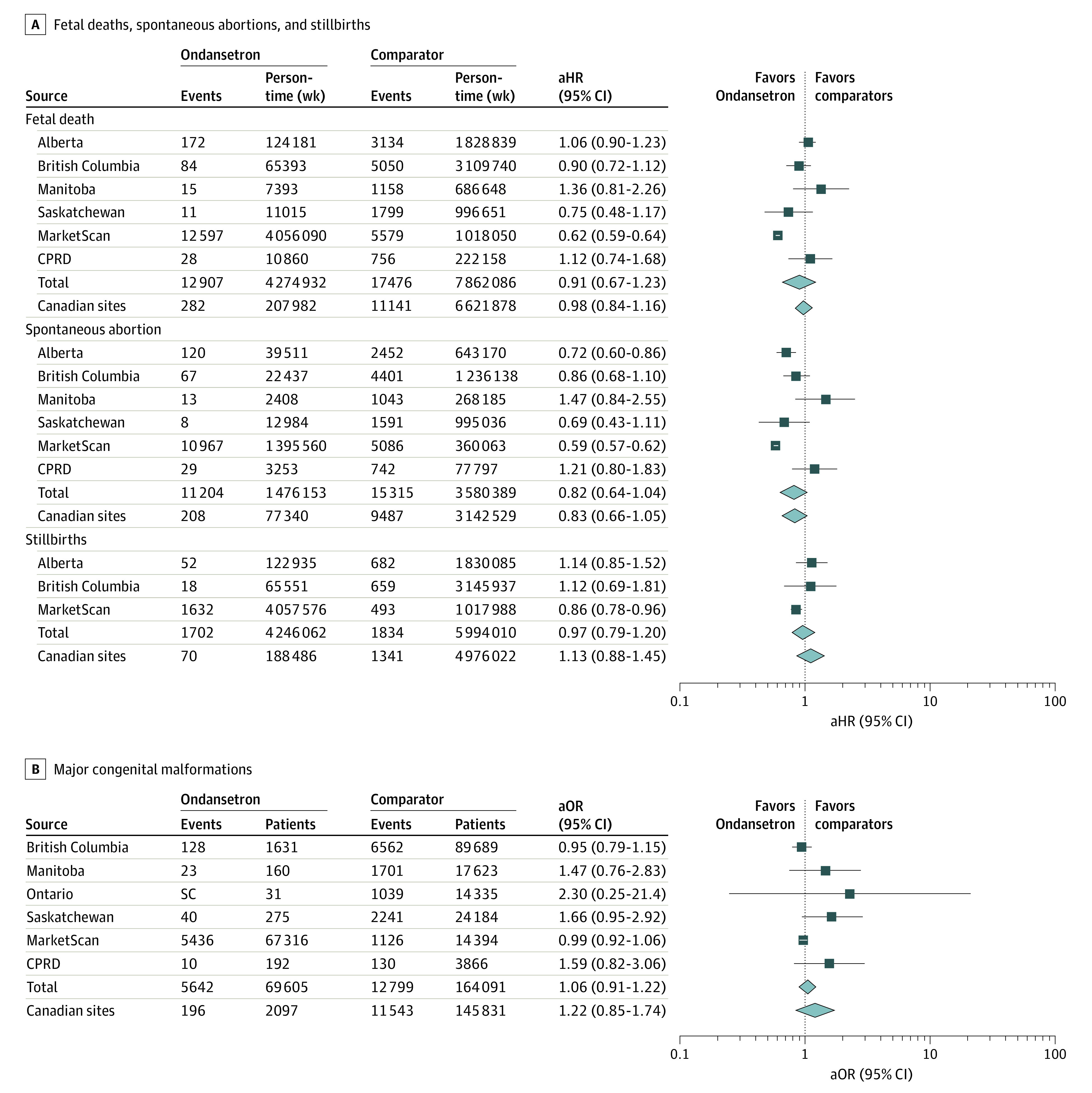
Analysis of Ondansetron Exposure and Occurrence of Adverse Fetal Outcome A, Adjusted hazard ratios (aHRs) are shown for fetal deaths, spontaneous abortions, and stillbirths. B, Adjusted odds ratios (aORs) are shown for major congenital malformations. CPRD indicates Clinical Practice Research Datalink; SC, small cell (ie, with ≤5 patients).

Our results were robust to the exclusion of the large MarketScan database and remained robust after sensitivity and subgroup analysis (Table 4). In general, restricting exposure assessment to the first trimester and to second-line exposures resulted point estimates closer to the null. The exception to these overall findings was the results for exposure during 4 to 10 weeks gestation, which suggested an increased risk of stillbirth in the Canadian databases (HR, 1.64; 95% CI, 1.01-2.66) and a protective association in the US data (HR, 0.52; 95% CI, 0.42-0.66). The analysis in the British Columbia database, which allowed for bidirectional switching between ondansetron and a comparator, yielded an HR for the primary outcome of 0.92 (95% CI, 0.71-1.18), which was close to the HR of 0.90 in the main British Columbia analysis.

**Table 4.** Sensitivity and Subgroup Analyses of Adverse Pregnancy Outcomes With Ondansetron vs Other Antiemetic Drugs

Exposure definition and subgroup	HR (95% CI)	
	All databases	Canadian databases only
Fetal death		
Exposed during pregnancy, first outcome of type for mother	1.05 (0.68-1.61)	1.21 (0.88-1.66)
Exposed during 4-10 wk gestational age		
Main analysis	0.84 (0.59-1.18)	0.79 (0.49-1.26)
First outcome of type for mother	1.08 (0.69-1.70)	1.13 (0.94-1.36)
Second-line or later exposure, main analysis	0.96 (0.69-1.33)	0.78 (0.57-1.07)
Stillbirth		
Exposed during pregnancy, first outcome of type for mother	0.96 (0.76-1.20)	1.15 (0.86-1.54)
Exposed during 4-10 wk gestation		
Main analysis	1.06 (0.43-2.62)	1.64 (1.01-2.66)
First outcome of type for mother	1.14 (0.41-3.17)	1.86 (1.03-3.36)
Second-line or later exposure, main analysis	2.11 (1.12-3.97)	2.01 (0.99-4.06)
Spontaneous abortion		
Exposed during pregnancy, first outcome of type for mother	0.90 (0.65-1.24)	1.04 (0.70-1.55)
Exposed during 4-10 wk gestational age		
Main analysis	0.89 (0.63-1.27)	0.83 (0.57-1.23)
First outcome of type for mother	1.10 (0.71-1.73)	1.09 (0.89-1.35)
Second-line or later exposure, main analysis	0.93 (0.63-1.36)	0.70 (0.48-1.01)
Major congenital malformations, OR (95% CI)		
Exposed during pregnancy		
First outcome of type for mother	0.99 (0.93-1.06)	1.07 (0.82-1.39)
Sibling analysis (British Columbia)	NA	0.82 (0.54-1.24)
Exposed during 4-10 wk gestational age		
Main	1.02 (0.90-1.16)	1.17 (0.83-1.64)
First outcome of type for mother	0.97 (0.89-1.04)	1.06 (0.83-1.35)
Second-line or later exposure, main analysis	0.99 (0.67-1.46)	0.96 (0.61-1.52)

Abbreviations: HR, hazard ratio; NA, not applicable; OR, odds ratio.

## Discussion

In this international, multicenter cohort study of more than 450 000 pregnancies exposed to an antiemetic medication, exposure to ondansetron was not associated with increased risk of fetal death, spontaneous abortion, stillbirth, or major congenital malformations. These findings were generally consistent across various sensitivity and subgroup analyses, such as exclusion of the large MarketScan database, and timing of exposure. The interpretation of our results is compatible with those of other studies by Pasternak et al,^4^ Danielsson et al,^5^ and Huybrechts et al,^6,9^ all of which reported no increase in risk of major congenital malformations overall, although Huybrechts et al,^6^ Zambelli-Weiner et al,^10^ Lemon et al,^13^ and Picot et al^11^ did report increases in the risk of orofacial clefts and cardiac malformations. In contrast, we did not observe an increased risk in cardiac malformations among pregnancies exposed to ondansetron; however, our analysis lacked sufficient power to examine other malformations.

As in the present study, the Danish study by Pasternak and colleagues^4^ investigated spontaneous abortion and stillbirth and found no harmful association with ondansetron therapy. Although the conclusion of no increased harm is the same in both studies, the meta-analysis HRs in our study were substantially closer to the null. There are at least 2 possible explanations for this. In addition to the studies being conducted in different populations, the Danish study used an unexposed reference group in its main analysis. We used a reference group of pregnancies exposed to other antiemetic medications. Patients exposed to other antiemetics may be more comparable to patients who use ondansetron than to those who do not use antiemetics. Improved comparability was apparent in a sensitivity analysis in the Danish study by Pasternak and colleagues,^4^ which found that ondansetron vs an antiemetic antihistamine yielded an association substantially closer to the null than the main analysis, which used nonexposure as a reference. A second possible explanation for results closer to the null could be our use of a time-dependent exposure definition, which we used to avoid immortal time bias. Unexposed patients are at risk of spontaneous abortion from the beginning of pregnancy, but patients are not at risk of a spontaneous abortion induced by ondansetron until after treatment is started, sometime later in pregnancy. Application of time-fixed exposure in the analysis leads to misclassification of event-free, unexposed person-time, potentially inducing a spurious survival advantage in the drug-exposed group.

### Strengths and Limitations

Our study has several important strengths. First, the use of ondansetron for NVP is rare in Canada. CNODES was able to combine data from multiple provinces and 2 international databases to obtain one of the largest studies thus far on the safety of ondansetron during pregnancy. Second, the broad capture of patients who used antiemetics for NVP from multiple countries also contributes to the generalizability of our results. Third, antiemetic therapy choices for NVP are subject to variable patient and physician preferences, local guidelines, and drug availability. This means that pregnancy studies cannot safely assume that the timing and duration of antiemetic exposures is nondifferential between drugs or between drug-exposed and nonexposed person-time. Use of time-dependent exposure modeling, therefore, also represented an important contribution of our study. Fourth, the use of a reference group of patients using other antiemetics minimized confounding by indication.

Several limitations of our analyses merit mention. The analysis of major congenital malformations lacked power to evaluate certain specific types of malformations, such as oral clefts and ventricular septal defects, for which concerns have been raised elsewhere.^6,10,11,12^ Although the number of specific types of malformations precluded precise comparison between ondansetron and the other antiemetics, the total number of pregnancies analyzed suggests that any such malformations attributable to ondansetron were exceptionally rare. Confounding by indication is a common threat to validity in studies of drug safety. Women who experience spontaneous abortion or have children with fetal malformations may have a reduced odds of NVP.^14,25^ To address confounding by indication as a source of bias, we adjusted our analyses for coded hospitalizations and ambulatory visits for nausea and/or vomiting. Furthermore, we used a reference group who received alternative antiemetic medications during pregnancy. Although we anticipate these measures successfully addressed confounding by indication, it remains a possibility that imperfect NVP diagnostic coding and variable antiemetic choice and efficacy resulted in some residual bias.

The stillbirth sensitivity analyses showed associations with ondansetron exposure during 4 to 10 weeks of gestation: harmful in Canada and protective in the US. These associations, which were closer to the null in the main analysis, are likely to be spurious and may be a consequence of selection bias from excluding women from the sensitivity analysis who used antiemetics before 4 weeks of gestation. In Canada, such patients were more likely to have used a reference antiemetic other than ondansetron, and in Canada ondansetron tends to be used after other antiemetics have been tried. Exclusion of patients in Canada using antiemetics in the first few weeks of pregnancy thus excluded more patients treated with a comparator than with ondansetron. The opposite was true in the US, where ondansetron is more likely to be used as first-line therapy for NVP.

Identification of some pregnancy outcomes in our claims databases was subject to misclassification. We assumed such misclassification was nondifferential between ondansetron-exposed patients and those who received an active comparator drug. However, this may be a strong assumption given the pattern of use of ondansetron as first-line therapy in the US and as second-line therapy in Canada. GA will also have been estimated with error because it was imputed in some instances in the Canadian data and in all records in the MarketScan database. Our databases generally lacked nonprescription and over-the-counter therapies used in pregnancy. Our inability to assess inpatient use of ondansetron, which comprised important proportions of exposures in the studies by Zambelli-Weiner et al^9^ and Lemon et al,^10^ could introduce immeasurable time bias by misclassifying exposure in some patients with severe NVP, thus diluting the association with ondansetron in studies comparing users with nonusers, unlike our study that used a comparator. We expect this posed minimal threat to the validity of our analyses because hospitalization with hyperemesis gravidarum occurs in approximately 1% of all pregnancies^26^ and because of evidence from Huybrechts and colleagues,^9^ who reported that intravenously administered ondansetron was not associated with an increase in the risk of cardiac malformations, oral clefts, or congenital malformations overall.

## Conclusion**s**

In this large, international, multicenter cohort study, there was no credible association between exposure to ondansetron during pregnancy and increased risks of fetal death, spontaneous abortion, stillbirth, or major congenital malformations compared with exposure to other commonly used antiemetic drugs.

## Notes

Supplement. **eAppendix 1.** Information on Study Databases **eAppendix 2.** Identification of Outcome Events **eAppendix 3.** Diagnosis and Procedure Codes Used to Define Outcomes **eAppendix 4.** Exposure Ascertainment Methodology
