# Supplementary material for: Comparison of Pregnancy Outcomes of Patients Treated With Ondansetron vs Alternative Antiemetic Medications in a Multinational, Population-Based Cohort
Source: JAMA Netw Open. 2021 Apr 23;4(4):e215329. doi: 10.1001/jamanetworkopen.2021.5329 (PMC8065380; doi:10.1001/jamanetworkopen.2021.5329)
Supplement: Supplement. — eAppendix 1. Information on Study Databases eAppendix 2. Identification of Outcome Events eAppendix 3. Diagnosis and Procedure Codes Used to Define Outcomes eAppendix 4. Exposure Ascertainment Methodology [file jamanetwopen-e215329-s001.pdf]

Supplemental Online Content

Dormuth CR, Winquist B, Fisher A, et al; Canadian Network for Observational Drug Effect Studies (CNODES) Investigators. Comparison of pregnancy outcomes of patients treated with ondansetron vs alternative antiemetic medications in a multinational, population-based cohort. *JAMA Netw Open*. 2021;4(4):e215329. doi:10.1001/jamanetworkopen.2021.5329

eAppendix 1. Information on Study Databases
eAppendix 2. Identification of Outcome Events
eAppendix 3. Diagnosis and Procedure Codes Used to Define Outcomes
eAppendix 4. Exposure Ascertainment Methodology

This supplemental material has been provided by the authors to give readers additional information about their work.

© 2021 Dormuth CR et al. *JAMA Network Open.*

eAppendix 1: Information On Study Databases

Site	Databases				
	**Population**	**Drug data and dispensing captured**	**Prescription drug claims**	**Hospitalization data**	**Outpatient billing data**
**British Columbia**	All (excludes ~4% of population which is federally insured)	Dispensing (All)	Healthideas Database: PharmaNet Claims History extract	Healthideas Database: Extract of CIHI Discharge Abstracts ICD-9: 1994-2002; ICD-10: Since 2002	Healthideas Database: Medical services plan fee for service billings (ICD-9)
**Alberta**	≥18 years	Dispensing (Public)	Pharmaceutical Information Network Dispenses	CIHI Discharge Abstract Database ICD-9: 1994-2002; ICD-10: Since 2002	Practitioner Claims (ICD-9)
**Saskatchewan**	All	Dispensing (Public)	Prescription Drug Plan Historical Claims	CIHI Discharge Abstract Database ICD-9: Until Mar 2002; ICD-10 Since Apr 2002:	Medical Services Branch (ICD-9)
**Manitoba**	All	Dispensing (All)	Drug Program Information Network (DPIN)	Hospital Abstracts User Manual Database (prior to April 1, 2004) Discharge abstract data/Manitoba Abstract Data Elements Database (since April 1, 2004) ICD-9: Apr 1979 to Mar 2004 ICD-10: Since Apr 2004	Manitoba Health Medical- Physician Services (ICD-9)
**Ontario**	Social assistance recipients	Dispensing (Public)	Ontario Drug Benefit Plan Database	CIHI Discharge Abstract Database ICD-9: Until Mar 2002; ICD-10: Since Apr 2002	Ontario Health Insurance Plan (OHIP) Claims History Database (Modified ICD-8)
**CPRD**	All	Dispensing (All)	General practitioner practice records linked to Hospital Episode Statistics hospitalization data and Office for National Statistics vital statistics information		
**IBM MarketScan® Commercial database**	Commercial Database: CCAET files. Enrollment data from large employers and health plans	Dispensing (Insured)	Commercial Database: CCAED files (Jan 2006 – Dec 2016)	Commercial Database: CCAEI files (Jan 2006 – Dec 2016)	Commercial Database: CCAEO files (Jan 2006 – Dec 2016)

© 2021 Dormuth CR et al. *JAMA Network Open.*

eAppendix 2: Identification of Outcome Events

The table below provides information on the eligible windows during pregnancy and exposure in which events were counted in the analyses of primary and secondary outcomes. Note: the term COHORTENTRY_GA was the gestational age (GA) in days when a study or comparator drug is first initiated, and OUTCOME_GA was gestational age on the date the outcome event occurred. These terms were not used in the manuscript text.

Table: Event Eligibility Windows

Event Type	Eligible Window for Event	Event Date
**Primary Outcome**		
Fetal Death<br>(stillbirth or spontaneous abortion)	≥COHORTENTRY_GA, until and including the OUTCOME_GA (gestational age in days on the OUTCOMEDT + 1 day	Date of event or birth
**Secondary Outcomes**		
Stillbirth	≥COHORTENTRY_GA, until and including the OUTCOME_GA	Date of birth
Spontaneous Abortion	≥COHORTENTRY_GA, until COHORTENTRYDT + 140 days, inclusive.	Date of event
Major Congenital Malformation	>COHORTENTRYDT	Date of birth

© 2021 Dormuth CR et al. *JAMA Network Open.*

eAppendix 3: Diagnosis And Procedure Codes Used To Define Outcomes

The following diagnostic, procedure and fee codes were used to identify outcome events. An ‘x’ denotes all codes with the stated prefix.

Stillbirth

ICD-9 Codes

ICD-9 Code	Description
V27.1x	Outcome of delivery, single stillborn
V27.4x	Outcome of delivery, twins, both stillborn
V27.7x	Outcome of delivery, other multiple birth, all stillborn
656.4	Intrauterine death, late

ICD-10-CA Equivalent Codes

ICD-10-CA	Description
O36.4	Intrauterine death, late
Z37.1x	Single stillbirth
Z37.4x	Twins both stillborn
Z37.7x	Multiples, all stillborn

Fee-for-Service Codes (in Saskatchewan, adapted for each province)

FFS Codes	Description
241P	Stillbirth

Spontaneous Abortion

ICD-9 Codes

ICD-9 Codes	Description
634.x	Spontaneous Abortion
630.x	Hydatidiform mole
631.x	Other abnormal products of conception
632.x	Missed abortion
633.x	Ectopic pregnancy
637.x	Unspecified abortion

© 2021 Dormuth CR et al. *JAMA Network Open.*

ICD-10-CA Equivalent Codes

ICD-10-CA	Description
O03.x	Spontaneous Abortion
O01.x	Hydatidiform mole
O02.x	Other abnormal products of conception
O05.x	Other abortion
O00.x	Ectopic pregnancy

Fee-for-Service Codes (in Saskatchewan, adapted for each province)

FFS Codes	Description
350P	Spontaneous abortion
48P, 248P	Ectopic gestation removal

Induced Abortion

ICD-9 Codes

ICD-9 Code	Description
635.x	Legally induced abortion
636.x	Illegal abortion

ICD-10-CA Equivalent Codes

ICD-10-CA	Description
O04.x	Medical abortion

Fee-for-Service Codes (in Saskatchewan, adapted for each province)

FFS Codes	Description
50P	Therapeutic/Medical abortion
250P	Therapeutic/Medical abortion

Termination of Pregnancy for Fetal Anomaly (TOPFA, included with induced abortions in the analysis)

TOPFFA included a code for induced abortion, co-occurring with one (or more) of the following during the same care episode (i.e., hospitalization or service date) as the induced abortion code:

© 2021 Dormuth CR et al. *JAMA Network Open.*

1. ICD-9 code 655.x or ICD-10 code O35.x
2. Livebirth
3. Stillbirth

<u>Live birth</u>

ICD-9 Codes

ICD-9 Code	Description
V27.0x	SINGLE LIVE BIRTH
V27.2x	TWINS, BOTH LIVE BORN
V27.5x	OTHER MULTIPLE BIRTH, ALL LIVE BORN

ICD-10-CA Equivalent Codes

ICD-10-CA	Description
Z37.0x	SINGLE LIVE BIRTH
Z37.2x	TWINS, BOTH LIVE BORN
Z37.5x	OTHER MULTIPLE BIRTH, ALL LIVE BORN

<u>Birth Type Mixed or Unspecified</u>

ICD-9 Codes

ICD-9 Code	Description
V27.3x	TWINS, ONE LIVE BORN AND ONE STILLBORN
V27.6x	OTHER MULTIPLE BIRTH, SOME LIVE BORN
V27.9x	UNSPECIFIED

ICD-10-CA Equivalent Codes

ICD-10-CA Code	Description
Z37.3x	TWINS, ONE LIVE BORN AND ONE
Z37.6x	OTHER MULTIPLE BIRTH, SOME LIVE BORN
Z37.9x	UNSPECIFIED

© 2021 Dormuth CR et al. *JAMA Network Open.*

Major Congenital Malformations

Major congenital malformations were defined using the European Surveillance of Congenital Anomalies (EUROCAT), International Clearinghouse for Birth Defects Surveillance and Research (ICBDSR), and Public Health Agency of Canada classifications, with some modifications (Pasternak et al, 2013).

1. We extracted all physician visits during the follow-up with diagnosis codes of congenital malformations, based on the list of codes in the table at the end of this section, column ‘A’.

2. We extract all hospitalizations with any diagnosis code of a congenital malformation (based on codes in the table below) and admission date. For records with ICD-9, we checked each diagnosis variable individually, and selected the record if the code was included in Column ‘B’ but not in Column ‘C’. For example, if the diagnosis code was 743.x, but not 743.6 or 743.8, then we included the malformation. If the code was 743.6 we excluded the malformation. We repeated this process using Columns ‘D’ and ‘E’.

Table: ICD Codes Used to Identify Malformations

**Category**	**Physician Services**	**Hospital Services**			
	**ICD-9: 3 digits**	**ICD-9: 4 digits**	***Exclusion for minor anomalies**	**ICD-10**	***Exclusion for minor anomalies**
Column	**A**	**B**	**C**	**D**	**E**
Nervous system	740-742	740.0–742.9		Q00.0–Q07.9	
Eye, ear, face and neck	743, 744	743.0-744.9	743.6, 743.8, 744.1-744.9	Q10.0-Q18.9	Q10.0-10.6, Q13.0, Q13.2, Q13.5, Q15.8, Q17.0-Q17.5, Q17.8, Q17.9, Q18.0-Q18.9
Circulatory system	745-747	745.0-747.9	747.0, 747.5	Q20.0-Q28.9	Q25.0, Q27.0
Respiratory	748	748.0-748.9	748.2, 748.3	Q30.0-Q34.9	Q30.2, Q30.8, Q31-Q32, Q33.1
Orofacial clefts	749	749.0-749.2		Q35.0-Q37.9	Q35.7
Digestive system	750, 751	750.0-751.9	750.0, 750.1, 750.2, 750.5, 750.6, 751.0, 751.5	Q38.0-Q45.9	Q38.1-Q38.6, Q40.0, Q40.1, Q43.0, Q43.4-Q43.9

© 2021 Dormuth CR et al. *JAMA Network Open.*

Category	Physician Services	Hospital Services			
	**ICD-9: 3 digits**	**ICD-9: 4 digits**	***Exclusion for minor anomalies**	**ICD-10**	***Exclusion for minor anomalies**
Column	**A**	**B**	**C**	**D**	**E**
Genital organs	752	752.0-752.9	752.4, 752.5, 752.8	Q50.0-Q56.9	Q52.2, Q52.3, Q52.4, Q52.5, Q52.6, Q52.7, Q52.8, Q53, Q54.4, Q55.1, Q55.2, Q55.6, Q55.8, Q55.9
Urinary system	753	753.0-753.9	753.6	Q60.0-Q64.9	Q61.0, Q62.7, Q63.3, Q64.2-Q64.3
Musculoskeletal system	754-756	754.0-756.9	754.0, 754.1, 754.7, 754.8, 756.0, 756.2	Q65.0-Q79.9	Q65.3-Q65.6, Q66.2, Q66.3, Q66.5-Q66.9 Q67.0-Q67.4, Q67.6-Q67.8, Q68.0, Q68.1, Q68.3-Q68.8, Q70.3, Q74.1, Q75.0, Q75.2, Q75.3, Q75.8, Q76.0, Q76.5, Q79.5, Q79.8
Integument	757**	757.0-757.9	757.2, 757.3, 757.4, 757.5, 757.6, 757.8	Q80.0-Q84.9	Q81, Q82.1-Q82.8, Q83.2, Q83.3, Q83.8, Q84.1, Q84.2, Q84.3, Q84.4, Q84.5, Q84.6, Q84.8
Chromosomal	758	758.0-758.8	758.4	Q90.0-Q99.2	Q95.0, Q95.1
Other	759	758.9, 759.0-759.9	759.9	Q85.0-Q89.9, Q99.8, Q99.9	Q89.9

Congenital malformations were excluded if they were on our list of excluded malformations defined in the following two tables.

Table: Specific Congenital Malformations linked to Medication Use

Category	Hospital Services			
	**ICD-9**	***Exclusion for minor anomalies**	**ICD-10**	***Exclusion for minor anomalies**
Isolated cleft palate	749.0		Q35.x	Q35.7
Ventricular septal defects	745.4		Q21.0	
Tetralogy of Fallot	745.2		Q21.3	
Hypoplastic left heart	747.7		Q23.4	

© 2021 Dormuth CR et al. *JAMA Network Open.*

Category	Hospital Services			
	**ICD-9**	***Exclusion for minor anomalies**	**ICD-10**	***Exclusion for minor anomalies**
Cardiac overall	745.x-747.2x	747.0	Q20.x-Q26.x	Q25.0
Diaphragmatic hernia	756.6		Q79.0	

Table Other Excluded Anomalies

	ICD-9	ICD-10
Anomalies with known causes	760.7	Q86
Chromosomal anomalies	758	Q90-99
Genetic syndromes	756.4, 756.3, 756.8, 759.5, 759.6, 759.8	Q75.1, Q75.4, Q77.1, Q77.2, Q79.6, Q85, Q87
Congenital virus infections	771.0, 771.1, 771.2, 771.2	P35.0, P35.1, P35.2, P371

ADDITIONAL NOTES ON CPRD:

Regarding the CPRD, we included pregnancies identified in the CPRD pregnancy register, which uses a validated algorithm (https://pubmed.ncbi.nlm.nih.gov/31197928/) to identify pregnancies from the general practitioner records. This register includes a unique identifier for the mother and pregnancy, the date of delivery, the estimated gestational age, and the pregnancy outcome. We used the 'Gold' version of the CPRD, as a pregnancy register is not yet available for CPRD AURUM. CPRD primary care records (including the pregnancy register) were linked to hospitalization data (acute patient care and maternity) from Hospital Episode Statistics (HES) and Office for National Statistics (ONS) vital statistics data, with inclusion restricted to linkable patients. Pregnancy outcomes were defined using the outcome variable in the CPRD pregnancy register. Congenital malformations were defined using diagnoses recorded in maternal and offspring CPRD and HES records, with the offspring identified using the Mother-Baby linkage. Comorbidities were defined using Read codes in CPRD primary care data and ICD-10 codes from HES, and prescription drug information was assessed using product codes and BNF codes recorded in general practitioner prescription records. ONS vital statistics data were used to supplement CPRD and HES data to define censoring.

For the CPRD analysis of congenital malformations, we used the CPRD Mother-Baby link to identify the offspring records in the CPRD. The Mother-Baby Link was created using a cartesian join of mothers to babies using the general practitioner practice identification number and the practice-specific family number, with inclusion restricted to records whose delivery date and estimated birth date are within 60 days.

© 2021 Dormuth CR et al. *JAMA Network Open.*

eAppendix 4: Exposure Ascertainment Methodology

For the main analysis, exposure was assessed as follows:

> Once a woman was exposed to ondansetron or a comparator antiemetic, she was considered exposed until the end of the follow-up (end of pregnancy).
>
> Once a woman started contributing person-time to the comparator antiemetic category, she could only subsequently switch her exposure status to ondansetron. If a woman tried another antiemetic after ondansetron, her exposure status remained defined as ondansetron-exposed.
>
> A woman could not contribute person-time to two different exposure categories at the same time.
>
> In rare instances where a woman started NVP pharmacotherapy simultaneously with ondansetron and a comparator antiemetic, she was assigned to the ondansetron-exposed category.

The table below provides information on the eligible windows during pregnancy during which exposure was assessed. Note: the term COHORTENTRYDT denotes the first day of pregnancy. This variable name was not used in the manuscript text.

Table: Exposure Assessment Windows

Event Type	Exposure Assessment Window<br>(Drug dispensed during window)
**Primary Outcome**	
Fetal Death (stillbirth or spontaneous abortion)	In order to enter cohort, exposed to a study and or comparator drug ≥COHORTENTRYDT, until, but not including, the outcome date<br>(Sensitivity analysis of exposure between 4 and 10 weeks: In order to enter cohort, exposed to a study and or comparator drug ≥COHORTENTRYDT + 27 days, until the earliest of COHORTENTRYDT + 69 days or up to and including the day before the outcome; but NOT exposed to a study and or comparator drug ≥COHORTENTRYDT and < COHORTENTRYDT + 27)
**Secondary Outcomes**	
Stillbirth	In order to enter cohort, exposed to a study and or comparator drug ≥COHORTENTRYDT, until outcome date, until, but not including, the outcome date<br>(Sensitivity analysis: In order to enter cohort, exposed to a study and or comparator drug ≥COHORTENTRYDT + 27 days, until the earliest of COHORTENTRYDT + 69 days, or up to and including the day before the outcome; but NOT exposed to a study and or comparator drug ≥COHORTENTRYDT and < COHORTENTRYDT + 27)
Spontaneous Abortion	In order to enter cohort, exposed to a study and or comparator drug ≥COHORTENTRYDT, until the earliest of COHORTENTRYDT + 140 days, or up to and including the day before the outcome

© 2021 Dormuth CR et al. *JAMA Network Open.*

	(Sensitivity analysis: In order to enter cohort, exposed to a study and or comparator drug ≥COHORTENTRYDT + 27 days, until the earliest of COHORTENTRYDT + 69 days, or up to and including the day before the outcome; but NOT exposed to a study and or comparator drug ≥COHORTENTRYDT and < COHORTENTRYDT + 27)
Major Congenital Malformation	In order to enter cohort, exposed to a study and or comparator drug COHORTENTRYDT to COHORTENTRYDT + 83 days, inclusive.<br>(Sensitivity analysis: In order to enter cohort, exposed to a study and or comparator drug ≥COHORTENTRYDT + 27 days, until COHORTENTRYDT + 69 days, inclusive)

© 2021 Dormuth CR et al. *JAMA Network Open.*
